# Comparative evaluation of the postbleaching application of sodium ascorbate, alpha‐tocopherol, and quercetin on shear bond strength of composite resin to enamel

**DOI:** 10.1002/cre2.655

**Published:** 2022-09-15

**Authors:** Marzieh Moradian, Maryam Saadat, Mohammad Hossein S. Shiri, Fatemeh Sohrabniya

**Affiliations:** ^1^ Department of Operative Dentistry, Oral and Dental Disease Research Center, School of Dentistry Shiraz University of Medical Sciences Shiraz Iran; ^2^ Student Research Committee, Department of Operative Dentistry, School of Dentistry Shiraz University of Medical Sciences Shiraz Iran

**Keywords:** alpha‐tocopherol, antioxidants, quercetin, sodium ascorbate

## Abstract

**Objective:**

This study aimed to evaluate and compare the impacts of the postbleaching application of sodium ascorbate, alpha‐tocopherol, and quercetin on the shear bond strength (SBS) of composite resin.

**Material and Methods:**

60 extracted intact maxillary first premolars were collected and were randomly divided into five experimental groups as follows (*n*=12): Group A (negative control): no bleaching, Group B (positive control): bleaching with 40% hydrogen peroxide (HP), Group C: HP±10% sodium ascorbate for 10min, Group D: HP±10% alpha‐tocopherol for 10min, and Group E: HP±1% quercetin for 10min. Composite bonding was done immediately after bleaching for Groups B‐E and without any treatment for Group A. After being stored in distilled water at room temperature for 24h, all specimens were tested for SBS in the universal testing machine. One‐way analysis of variance and Tukey's post‐hoc test were used to analyze the SBS values of all groups.

**Results:**

The results showed that the bonding of composite to the unbleached group exhibited the highest mean value of SBS (22.68±2.91MPa). Among the antioxidant‐treated groups, the highest SBS value was detected in quercetin‐treated specimens (15.45±1.58MPa), which was significantly different from the positive control group (*p*<.05).

**Conclusion:**

It could be concluded that 10% quercetin applied for 10min increased the bond strength to bleached enamel, but it was not able to reverse it completely.

## 1 INTRODUCTION

Tooth discoloration has been one of the most prominent esthetic concerns in dentistry, which is arisen by multiple extrinsic and intrinsic factors. Extrinsic dental staining is usually produced by tobacco smoking, dental plaque, bacteria, food, beverages, and some medications. However, intrinsic dental staining is predominantly caused by dental caries, medications, trauma, genetics, and aging. Extrinsic stains can be eliminated via dental prophylaxis. If tooth staining is still obvious after the elimination of extrinsic staining, external tooth whitening can be suggested (Janjua et al., 2022; Jurema et al., 2018).

Bleaching materials mostly include carbamide peroxide and hydrogen peroxide, which have been widely used successfully (Vidhya et al., 2011; Wijetunga et al., 2021; Y. Xu et al., 2018). There are some adverse effects associated with dental restorative materials, such as pulp sensitivity, release of the selected components, and alteration of the enamel surface (Goldberg et al., 2010). One of the most critical problems with bleaching materials is the compromised composite resin's bond strength to enamel immediately after the bleaching process. Strategies intended to eliminate the decreased bond strength include treating the enamel with alcohol, removal of the superficial enamel layer, using adhesive‐containing organic solvents, and the use of antioxidant agents (Arumugam et al., 2014; Cvitko et al., 1991; Kadiyala et al., 2015).

Both internal and superficial weakening of the bond has been reported as well. This can be due to the presence of residual peroxide and its interference with the resin tag formation and the inhibition of resin polymerization (Gökçe et al., 2008; Shamsedin et al., 2017; Titley et al., 1991). This decrease can be avoided by delaying restorative treatments by 1 day up to 3 weeks after tooth bleaching, which is the most usual approach in clinics (Kılınç et al., 2016). In this approach, residual peroxides release gradually. Sometimes, however, it is necessary to shorten this time for various reasons, including patients’ distant living places or lack of time for special events. Hence, researchers are looking for a material that scavenges free radicals to prevent them from disrupting the bonding procedure. In this context, it has been proposed to use a range of antioxidant agents, such as 10% sodium ascorbate, vitamin E, and proanthocyanidin following bleaching and before restoration (Gökçe et al., 2008; Kaya et al., 2008; Lai et al., 2002; Leandrin et al., 2020; Olmedo et al., 2021; Vidhya et al., 2011; Y. X. Xu et al., 2021).

Ascorbic acid (vitamin C) is an essential nutrient found in various foods, which has a vital role in collagen synthesis (Darr et al., 1993) and iron absorption (Lane & Richardson, 2014). Sodium ascorbate is one of the mineral salts of ascorbic acid, which is widely used for its antioxidant activity (Kowalczyk et al., 2018). Many studies have investigated its effect on the shear bond strength (SBS) of composite resin to bleached enamels (Kavitha et al., 2016; Sasaki et al., 2009; Türkmen et al., 2016).

Alpha‐tocopherol is the most prevalent form of vitamin E, which is found in a variety of tissues. It has antioxidant properties and can prevent chronic diseases and cancer (Yang et al., 2012). Alpha‐tocopherol has a significant role in protecting against free‐radical reactions that can cause various conditions, such as aging, cancer, circulatory diseases, arthritis, Alzheimer's disease, and respiratory diseases induced by pollution (Burton & Ingold, 1989). Vitamin E has an antioxidant activity, which can be attributed to four tocotrienols (alpha, beta, gamma, and delta) and tocopherols (Kavitha et al., 2016). Recent studies have indicated that alpha‐tocopherol may improve bond strength after bleaching (Dhingra et al., 2017; Ghaleb et al., 2020; Leandrin et al., 2020; Lokhande et al., 2018; Porto et al., 2021; Srivastava & Yeluri, 2021; Whang & Shin, 2014). By scavenging free radicals and molecular oxygen, vitamin E reverses bond strength. This mode of action is very similar to that of sodium ascorbate (Yang et al., 2012).

Flavonoids such as quercetin are found in many foods, fruits, and vegetables. They are also available as food supplements. Several benefits have been associated with quercetin, such as anticancer, anti‐inflammatory, and antioxidant properties (Porto et al., 2021). Quercetin can be potentially used abundantly for reversing the effects of bleaching on SBS (Shamsedin et al., 2017).

The present study aims to evaluate and compare the effects of using sodium ascorbate, alpha‐tocopherol, and quercetin on reversing the decreased SBS of composite resin to bleached enamels. The null hypothesis of this study is that using antioxidant solutions will not influence the SBS.

## 2 MATERIALS AND METHODS

**Figure 1:**
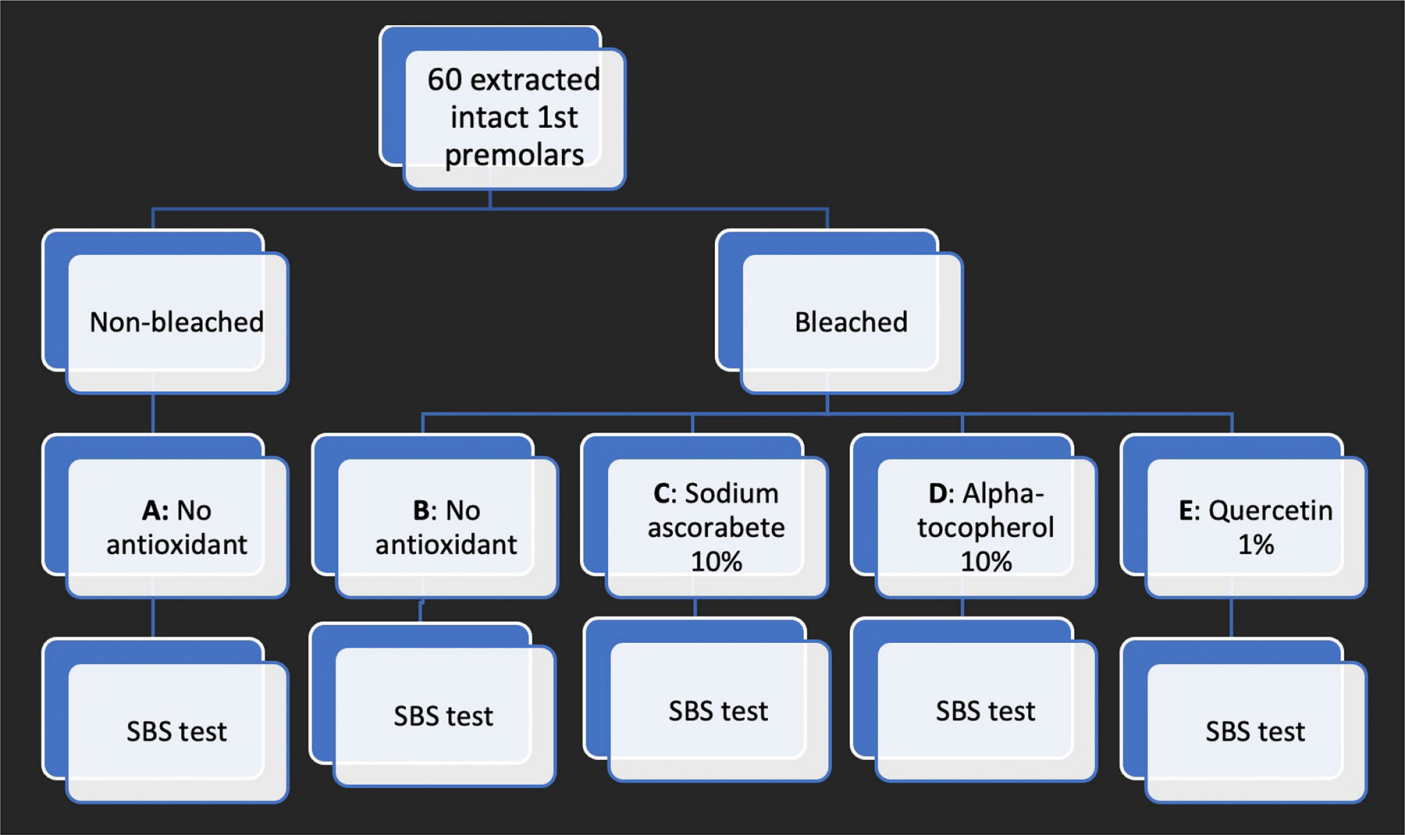
Diagram of the study design

The diagram of the study design has been depicted in Figure 1.

### 2.1 Specimen preparation

In this in‐vitro study, according to the guidelines provided by the Research Ethics Committee of Shiraz University of Medical Sciences (IR.SUMS.DENTAL.REC.1398.012), 60 extracted intact human maxillary first premolars were collected. The teeth were stored in 0.5% chloramine solution at 4°C for less than 2 months before use. The teeth were fixed in cylindrical acrylic resin blocks (2.5 cm in height and 2 cm in diameter) from the cementoenamel junction (CEJ), such a way that the coronal portions of the teeth were out of the blocks. The enamel surfaces of the specimens were wet‐polished with 600‐grit silicon carbide paper in a low‐speed handpiece with constant water coolant (Kaya et al., 2008). The prepared enamel surfaces of 48 teeth were bleached with 40% hydrogen peroxide gel (Opalescence, Ultradent Product Inc.) with two 20‐min applications according to the manufacturer's instructions. Then, the gel was rinsed off thoroughly with tap water.

### 2.2 Treatments

Totally, 12 teeth that were not bleached were separated for Group A (negative control) and the rest of the teeth were randomly divided into four groups (*n* = 12). In Group B (positive control), the teeth were only bleached with no antioxidant treatment. In Group C, 10 g of sodium ascorbate powder (Sigma‐Aldrich) was dissolved in 100 ml distilled water in a standard flask to obtain a 10% sodium ascorbate solution. In Group D, a 10% alpha‐tocopherol solution was prepared by dissolving 10 g of alpha‐tocopherol gel (Sigma‐Aldrich) in 100 ml of ethyl alcohol in a standard flask. In Group E, a 1% quercetin solution was prepared by dissolving 1 g of quercetin powder (Sigma‐Aldrich) directly into pure ethanol under water‐bath heating at 37°C. Immediately after bleaching, the enamel surfaces were exposed to antioxidant solutions for 10 min. Every minute, the solution was applied to the specimen with a syringe and was refreshed by microbrush to keep the enamel surface wet. Then, the specimens were rinsed with water for 30 s and were air‐dried.

### 2.3 Restorative procedure

Etching by 37% phosphoric acid gel (Denfil) for 20s was done for all the bleached surfaces. Afterward, they were rinsed with water spray for 20 s followed by the application of Adper single bond (3M ESPE; dental products), and were light cured by an LED unit (Demi plus) at a light intensity of 1200 W/cm^2^ for 20 s. Finally, composite resin (Filtek Z 250; 3M ESPE) restoration procedure was carried out by using a plastic mold measuring 3 mm in diameter and 2 mm in height and was light cured by an LED light‐curing unit for 20 s. All the specimens were stored in distilled water at room temperature for 24 h. All the procedures were done by one operator and at the same time for the five experimental groups.

### 2.4 SBS testing

The specimens were tested in shear mode using a chisel‐shaped blade of a universal testing machine (Zwick Roell, Z020, Germany) at a crosshead speed of 1mm/min. The force at failure was measured in Newton (N). Then, the SBS values were recorded in MPa. SBS was estimated in MPa by the following equation:

$$\mathrm{Bond}\mathrm{strength}\;(\mathrm{MPa})=\frac{\text{Peak force }(\text{newton})}{\text{area }(m^{2})}.$$

### 2.5 Statistical analysis

SPSS 25 was used to analyze the collected data. Data normality was assessed by the Shapiro–Wilk test. Then, one‐way analysis of variance (ANOVA) was used to compare the study groups according to SBS.

## 3 RESULTS

The results of ANOVA showed a significant difference among the study groups regarding SBS (*p* < .001). The mean SBS values in all five groups have been presented in Table 1. Accordingly, bonding of the composite to the unbleached group exhibited the highest mean of SBS (22.68 ± 2.91 MPa). Among the antioxidant‐treated groups, the highest SBS value was detected in the quercetin‐treated samples (15.45 ± 1.58 MPa). However, no significant difference was observed among Groups C, D, and E. Tukey's test showed a significant difference between Groups B (positive control) and E (quercetin) in this regard (*p* < .05).

**Table 1:** The mean and standard deviation of shear bond strength (MPa) in each group

Groups	Mean SBS ± SD
Group A (negative control)	22.68 ± 2.91^A^
Group B (positive control)	12.29 ± 2.20^C^
Group C (sodium ascorbate)	14.56 ± 2.72^BC^
Group D (alpha‐tocopherol)	12.67 ± 2.50^BC^
Group E (quercetin)	15.45 ± 1.58^B^

*Note*: The mean values followed by different uppercase letters indicate significant differences in the column (*p* < .05).

## 4 DISCUSSION

Using antioxidants appears to be the best, fastest, and most convenient way of reversing the decreased bond after bleaching (Leandrin et al., 2020; Olmedo et al., 2021; Pooja et al., 2021; Rodríguez‐Barragué et al., 2021; Shamsedin et al., 2017; Y. X. Xu et al., 2021). The results of the present study demonstrated that none of the antioxidant solutions reestablish the compromised bond strength in the bleached enamel. Thus, the null hypothesis was accepted.

In this study, sodium ascorbate was used as the gold standard, because it has been well evaluated in the literature (De Carvalho et al., 2016). Although the results indicated that sodium ascorbate increased the SBS, the difference between groups B and C was not statistically significant. In the same line, Tabatabaei et al. disclosed that sodium ascorbate was not effective in reversing the SBS after bleaching (Tabatabaei et al., 2011). They used 10% sodium ascorbate for 5 and 10 min. They revealed a slightly higher effect of sodium ascorbate after 10 min. Hence, the time of sodium ascorbate application may be important for its antioxidant effect and improvement of the SBS after bleaching. Kaya et al. (2008) also assessed the effect of the time of 10% sodium ascorbate application and demonstrated that sodium ascorbate became effective in the enamel surface after 60 min. Similar results were also obtained by Dabas et al. (2011) and Subramonian et al. (2015). In contrast, the findings of the research by Kadiyala et al. (2015) indicated that the use of 10% sodium ascorbate significantly increased SBS. In that study, carbamide peroxide was used as the bleaching agent. In the present study, however, 40% hydrogen peroxide was used. It is more potent than carbamide peroxide and generates more residual oxygen molecules, which can reduce SBS significantly.

Vitamin E refers to a group of tocopherols and tocotrienols, among which alpha‐tocopherol has the highest biological activity (Brigelius‐Flohé & Traber, 1999). In the current study, although alpha‐tocopherol slightly improved the SBS compared to the positive control group, its negligible effect was not statistically significant. Controversial results have been obtained in this regard in previous studies. The discrepancy among the results may be related to different times of application or different materials used for the bleaching process. For example, Kavitha et al. (2016) used 10% alpha‐tocopherol and it was the weakest antioxidant that could not increase the SBS significantly, which was in agreement with the current study results. This may be associated with the nonaqueous nature of this antioxidant, which impairs the polymerization process (Gogia et al., 2018). In another study by Thapa et al. (2013), the efficacy of alpha‐tocopherol 10% was time‐dependent. Accordingly, the best performance was detected at 60 min, but it was not efficient after 10 min, which justifies the results of the current investigation. In contrast, Bansal et al. (2019) revealed the significant effect of alpha‐tocopherol 10% on SBS reversal. It is important to note that carbamide peroxide was used as the bleaching agent, which is weaker than hydrogen peroxide. Thus, it seems that alpha‐tocopherol may be efficient in the reversal of SBS in bleached dentin if it is applied for a long or is used after bleaching with carbamide peroxide. Hence, the effect of vitamin E is recommended to be studied at different application times.

Quercetin is a suggested material for improving bond durability because of its good properties, including inhibition of reactive oxygen species (ROS) and cyclooxygenase (COX‐2) production, leading to the inhabitation of matrix metalloproteinases (MMPs) (Li et al., 2017; Lu et al., 2018; Porto et al., 2018). In addition, it is an antimicrobial material against Gram‐positive and ‐negative bacteria and viruses (Delnavazi et al., 2017; Kaul et al., 1985; Li et al., 2017). These properties make quercetin a useful agent for preventing recurrent carries. Like most antibacterial materials, quercetin is released in two stages: (1) the burst phase in the first few days, during which a high concentration of antioxidants is released and (2) the tail release phase after some weeks when the concentration of antioxidants is lower than the efficacy level. Nevertheless, quercetin is hardly soluble. It has been assumed that it cannot be washed away by saliva. Therefore, it may be used as a long‐term antioxidant‐releasing material (Li et al., 2017). Furthermore, quercetin can be beneficial because of its high antioxidant activity until 2 weeks after bleaching. Some studies have indicated the efficacy of quercetin in preventing bond degradation in dentin‐resin interface (Gotti et al., 2015; Moreira de Freitas Guimarães et al., 2021). Since few studies were conducted on quercetin and its effects on the reversal of SBS after bleaching, it was used in the present study to compare its antioxidant effects to other antioxidants mentioned above. In this study, a 1% concentration of quercetin was chosen to achieve optimal antioxidant performance. The results showed that the application of 1% quercetin solution for 10 min could reverse the SBS immediately after bleaching, and this increase was significant compared to the positive control group. A prior study by Shamsedin et al. (2017) demonstrated that different amounts of quercetin (0.1%, 0.5%, and 1%) could improve SBS to normal levels, regardless of the duration of application (5 and 10 min), which was consistent with the current study findings. Although quercetin increased the SBS in the present investigation, the SBS values were significantly lower in Group E (quercetin) compared to the unbleached group (negative control). In the abovementioned study, dimethyl sulfoxide (DMSO) was used as the solvent for quercetin, while ethanol was used in the current research. Besides, 15% carbamide peroxide was used as the bleaching agent in the former study, while 40% hydrogen peroxide was utilized in the present one. These may account for the differences between the two studies.

In the present study, no significant increases in mean SBS values were achieved in all groups after antioxidant application. Our results indicate that the mean SBS values of the specimens exposed to antioxidants were lower than those in the negative group.

This study had some limitations. The results of this study cannot be necessarily generalized to other brands of the used materials. One study (Porto et al., 2021) reported the dose‐dependent cytotoxic effect of quercetin solution on human dental pulp cells (HDPs), an increase in its concentration is accompanied by an increase in its cytotoxicity. The study indicated that it was difficult for quercetin to penetrate into the long dentinal tubules because dentinal tubules have an outward pressure in in‐vivo applications due to the outflow of dentinal fluid, eventually exerting cytotoxic effects on HDPs. Moreover, further studies are needed for determining the optimum concentration and duration of application of these materials. Higher concentrations of this flavonoid may have even faster and greater effects. Furthermore, using a different solvent like DMSO and dimethyl formamide (DMF) for quercetin may increase SBS after bleaching.

## 5 CONCLUSION

The results of this in‐vitro study demonstrated that bleaching significantly reduced the SBS of the resin composite to the bleached enamel. Hence, it could be concluded that the application of antioxidants onto bleached enamels before the bonding procedure did not completely neutralize the deleterious effects of bleaching.

## AUTHOR CONTRIBUTIONS

Marzieh Moradian and Maryam Saadat conceived the presented idea and developed the theory. Marzieh Moradian, Maryam Saadat, and Mohammad Hossein S. Shiri carried out the experiment and contributed to the final version of the manuscript. Marzieh Moradian and Maryam Saadat supervised the project. Mohammad Hossein S.i Shiri and Fatemeh Sohrabniya performed the analytic calculations and performed numerical simulations.

## CONFLICT OF INTEREST

The authors declare no conflict of interest.

## Data Availability

The data that support the findings of this study are available on request from the corresponding author.
